# Mild cognitive impairment history and current procedures in low- and middle-income countries: a brief review

**DOI:** 10.1590/1980-57642021dn15-020001

**Published:** 2021

**Authors:** Larissa Hartle, Helenice Charchat-Fichman

**Affiliations:** 1 Department of Psychology, Pontifícia Universidade Católica do Rio de Janeiro, Rio de Janeiro, RJ, Brazil.; 2 Department of Philosophy, Social, Human and Education Sciences, Università degli Studi di Perugia, Perugia, Italy.

**Keywords:** mild cognitive impairment, aging, activities of daily living, cognition disorders, comprometimento cognitivo leve, envelhecimento, atividades cotidianas, distúrbios cognitivos

## Abstract

Mild cognitive impairment (MCI) is a widely studied concept that has changed over time. Epidemiology, diagnosis, costs, prognostics, screening procedures, and categorization have been extensively discussed. However, unified guidelines are still not available, especially considering differences between low- and middle-income countries (LMIC) and high-income countries (HIC).

**Objectives:**

To contextualize and identify the main areas under investigation regarding MCI diagnosis and to investigate how much of the current knowledge is compatible with the diagnosis in an LMIC.

**Methods:**

This brief review followed the framework outlined for a scoping review and goes through the history of MCI and its diagnosis, the differences and relevance of LMIC research regarding the concept, and the current criteria for diagnosis.

**Results:**

Results show that the unique characteristics of LMIC influence the development of cognitive decline and how suitable procedures suggested by HIC can be used by LMIC to identify pathological aging processes in their early stages.

**Conclusion:**

Neuropsychological assessment of activities of daily living performance, considering the difference between omission and commission errors, is a more accessible course of action as a screening procedure for cognitive decline in LMIC.

## RESUMO.

O comprometimento cognitivo leve (CCL) é um conceito amplamente estudado que mudou ao longo do tempo. Epidemiologia, diagnóstico, custos, prognósticos, procedimentos de triagem e categorização já foram amplamente debatidos. No entanto, diretrizes unificadas ainda não estão disponíveis, especialmente considerando as diferenças entre países de baixa e média renda (PBMR) e países de alta renda (PAR).

**Objetivos:**

Contextualizar e identificar as principais áreas de pesquisa em relação ao diagnóstico do CCL e investigar quanto do atual conhecimento é compatível com o diagnóstico em PBMR.

**Métodos:**

Esta revisão se guiou a estrutura delineada para uma *scoping review*, percorrendo a história do CCL, o diagnóstico, as diferenças e a relevância da pesquisa de PBMR para o conceito e os critérios atuais para diagnóstico.

**Resultados:**

Os resultados mostram que as características únicas dos PMBR influenciam o declínio cognitivo e como os procedimentos sugeridos por PAR podem ser usados por PBMR para identificar processos de envelhecimento patológico em seus estágios iniciais.

**Conclusão:**

A avaliação neuropsicológica de atividades de desempenho de vida diária, considerando a diferença entre erros de omissão e comissão, é um curso de ação mais acessível como um procedimento de triagem para declínio cognitivo em PMBR.

## INTRODUCTION

The latest United Nations World Population Aging Reports^1^ ^,^ ^2^ raised important issues about global aging. The global population aged 60 or older more than doubled from 1980 to today. The expectation is that it will double again by 2050, reaching over two billion and surpassing the number of children and teenagers aged between 10 and 24. According to the 2017 report, life expectancy has increased more than twenty years globally since 1950 - reaching more than 70 years old. By 2050, the report estimates that global life expectancy will exceed 80 years old in Europe, Latin America, and Oceania. In Africa and Asia, it is expected to surpass 70 years old. The numbers are growing faster in developing countries, where 80% of older adults are expected to be living by 2050.

Different pathological conditions are related to aging. The cognitive decline caused by neurodegenerative disorders is one of them.^3^ Such disorders cause extensive disability in the long term, and no or few treatments are available. For this reason, these disorders, grouped under the term dementia, today are a considerable economic and public health challenge.^3^ Worldwide, the global costs of dementia are increasing and went from US$ 604 billion in 2010 to US$ 818 billion in 2015.^4^ In 2017, the mean value of care was estimated to be US$ 321,780 per person with dementia, more than two times the health expenses of older adults without the condition.^5^

If some decades ago it was enough, regarding neurodegenerative disorders, to differentiate between normal aging and dementia, today early detection is the goal to enable early interventions.^6^ In this context, mild cognitive impairment (MCI) increases as a relevant concept. MCI is seen as a preclinical stage of dementia.^7^ Although there are other prognostics,^8^ people diagnosed with MCI have a higher rate of dementia development than those not diagnosed.^9^ Of diagnosed dementia cases, 75% are Alzheimer’s disease (AD).^7^ Population studies that indicate the prevalence of MCI in the population vary significantly in their results due to different diagnostic criteria, differences between prospective and retrospective analyses, and according to which MCI subtypes are being sought.^10^ The estimate of older adults with MCI ranges from 12 to 18% globally,^6^ although some studies reach percentages as high as 42% in particular countries.^11^

There are fewer data available in low- and middle-income countries (LMIC), although dementia increase is more prominent in those places.^7^ ^,^ ^12^ In Latin American countries (LAC), the reported prevalence of dementia varies between 2 and 13.7%.^12^ Usually, there are more MCI cases than dementia,^11^ so a number higher than this should be expected. In Brazil, one study reported an incidence rate per 1000 person-years for MCI of 13.2, whereas globally the same incidence is usually between 8.5 and 31.9%.^13^ Direct and indirect costs of dementia in Brazil are estimated to be more than US$ 16,000 per patient annually,^14^ so any percentage becomes a challenge not only to individuals and their families but also to society.

Even so, most of the current discussion about MCI comes from the landscape in high-income countries (HIC).^12^ Thus, it is imperative to (1) contextualize and identify the main areas under investigation regarding MCI diagnosis and (2) investigate how much of the current knowledge is compatible with the diagnosis in an LMIC context, which were the aims of this brief review.

## METHODS

This brief review followed the framework outlined for a scoping review proposed by Arskey and O’Malley^15^ with some modifications regarding the analysis of the results. It can be divided into two parts. First, with the object of mapping the main areas where research about MCI is being done, an initial literature search was conducted. The terms (mild cognitive impairment) AND diagnosis AND ((cognition) OR (neuropsychological assessment) OR (functionality) OR (activities of daily living)) were used in PubMed to retrieve data from two resources from the last five years: MEDLINE and PubMed Central. Given the overwhelming number of results, they were briefly analyzed until new results were considered superfluous to define the areas of interest useful to address the main research question. This first search defined this review article’s initial structure, i.e., Historic, Diagnosis, Cognition performance, and Activities of daily living sections.

After the first search, a second search with the same keywords was conducted regarding the Scielo database to retrieve data on LMIC. Results were analyzed in the same way, but this time regarding their fit to the sections already defined. As advised by Arksey and O’Malley,^15^ we also checked the bibliography of all studies included and hand-searched key journals. After identifying the relevance of each topic to address LMIC’s context influencing diagnosis, the Cognition section was merged with the Diagnosis section, and the Activities of daily living section was divided into Activities of daily living assessment, Activities of daily living performance awareness, and Types of errors in activities of daily living performance.

## HISTORIC

The term MCI was used for the first time by Reisberg et al. in 1982.^16^ It referred to the third stage on a scale of seven stages regarding AD progression. At that time, it was characterized mainly by a decline in memory capacity. The scale, named the Global Deterioration Scale, goes from a typical aging profile to severe dementia, where all basic activities of daily living (ADL) and verbal and psychomotor behaviors are compromised.^16^ In the same year, other authors also named *mild dementia* as the third of five stages of Alzheimer’s progression.^17^ This scale was called the Clinical Dementia Rating (CDR). Morris^18^ described the same condition ten years later. This scale resembles the current MCI concept but is not identical to it.^10^

It was Petersen who in 1999 refined the concept and started its current use.^6^ ^,^ ^10^ Regarding this definition, MCI was first described as the initial stage of AD. It was characterized predominantly by a decline in memory more than that expected for a specific age but not yet sufficient for the diagnosis of dementia.^19^ In 2004, the diagnostic entity of MCI was expanded. It went from being a stage of AD marked by memory decline to (1) embrace other cognitive domains and (2) be associated with other etiologies besides AD. Some examples are cognitive impairment related to vascular disease, frontotemporal and Lewy body dementia, and sleep and mood disorders.^6^ On the basis of this new definition, MCI was divided into four subtypes: (1) amnestic single-domain; (2) amnestic multiple-domain - when there is memory decline concomitantly with decline in other functions; (3) non-amnestic single-domain; and (4) non-amnestic multiple-domain - when there is no memory decline, but there are one or more compromised functions other than memory.^6^

But studies using different criteria lead to distinct classifications.^20^ Other categories have been proposed mostly because there is no clear and specific guideline to identify MCI.^21^ Criteria have been modified over time and have not yet reached a consensus.^8^ Nevertheless, the American Academy of Neurology concluded from an evidence-based medical review that the construct of MCI is of great clinical utility given the higher rate of conversion of diagnosed individuals to dementia compared to undiagnosed individuals.^22^ Besides that, the Diagnostic and Statistical Manual of Mental Disorders also incorporated similar concepts. The fourth version of the “Diagnostic and Statistical Manual of Mental Disorders (DSM-IV)” mentioned *Age-Related Cognitive Decline* in the section “Other Conditions That May Be a Focus of Clinical Attention”. On the other hand, DSM-V incorporated *Mild Neurocognitive Disorder* in the neurocognitive disorders chapter, with diagnostic criteria similar to MCI.^6^

## DIAGNOSIS

Although there are no unified guidelines to diagnose MCI, today neuropsychological assessment is seen as a useful tool to detect the condition.^8^ Different options have similar diagnostic accuracy,^23^ but brief options seem to primarily identify only the amnestic subtype, whereas other types would need a more comprehensive battery.^24^ Studies using the Mini-Mental State Examination (MMSE) as a gold standard show MoCA as the best tool to screen for MCI,^25^ although there are recommendations not to use MMSE as a measure for comparison.^23^ In Argentina, MoCA was found to have good accuracy in identifying both MCI and dementia, but it is influenced by educational level.^26^ In fact, more comprehensive analyses show MoCA as more suitable for screening for dementia than for MCI.^27^ ^,^ ^28^ In Brazil, one study also found that MoCA is the most recommended tool to screen for dementia, but it is not so accurate when screening for cognitive decline before a dementia diagnosis or for low educational levels.^29^

In these scenarios, characterized by a population with a more diverse culture and education background, other options seem to have advantages but are better for detecting dementia than MCI.^25^ ^,^ ^30^ Analyses comparing medical diagnosis to the assessment of processing speed measures reached similar results.^31^ Therefore, there is still room for discussion on how to detect MCI, especially considering its heterogeneity and considering the different contexts seen in LMIC and HIC.

For example, another way of diagnosing MCI in HIC is the use of biomarkers. HIC have been using biomarkers in research and clinical settings, but the approach seems to be restricted to those countries and some upper-middle-income countries.^12^ Biomarkers are measurable biological indicators of a physical condition.^7^ Examples are analysis of tau and phospo-tau protein, cerebrospinal fluid, beta-amyloid, fibrillar AB burden, and brain imaging.^8^ ^,^ ^21^ Current studies indicate that tau protein levels and beta-amyloid in the brain can be handy not only to detect MCI, but also to define its etiology.^6^ Biomarkers can also help determine the likelihood of progression from MCI to AD^8^ and even detect cognitive alterations before MCI occurs.^21^ Although this would make a preclinical diagnosis possible, the biomarkers’ clinical utility and benefits have also been questioned.^12^ ^,^ ^21^ The method is accurate and valued in research settings, but the cost is high,^21^ and most LMIC do not have access to the equipment needed.^12^

Due to all these disadvantages and difficulties, neuropsychological assessment is still a more reasonable approach to detect MCI, especially in LMIC.^12^ Considering the original criteria proposed by Petersen,^6^ the diagnosis is made on the basis of a cognitive decline complaint verified by clinical analyses, such as neuropsychological assessment. It must be determined whether there are one or more cognition areas affected, and the neuropsychological assessment is extremely useful to do so.^32^ According to the original criteria, ADL should also be analyzed. They should be preserved in MCI to make a differential diagnosis between this condition and dementia.

Although these conventional criteria exist, there is no unified approach to MCI diagnosis,^8^ and different cutoff points are used to define when a cognitive domain is impaired.^33^ Besides that, these criteria cover a broad spectrum of heterogeneous profiles and can present themselves as the initial stage of different dementias and affect other cognitive domains. Individuals with amnestic MCI single domain, for example, have an increased risk for developing AD. On the other hand, attention, concentration, and visuospatial deficits may indicate Lewy body dementia. Behavioral changes, inappropriate behavior, and executive problems can indicate a dysexecutive MCI and possible frontotemporal dementia.^6^ There is, however, also a possibility of staying stable or improving after an MCI diagnosis.^34^ Moreover, data from autopsy and imaging studies reveal that mixed pathologies are common.^8^

Moreover, there are still other factors that can affect MCI categorization, and education is one of them.^21^ This is crucial when thinking about how MCI and dementia affect LMIC, where access to education is not the same as in HIC. A Brazilian study found a 12.7% higher rate of MCI among illiterates. An analysis of Brazilian older adults without dementia showed that education was the best predictor of MMSE total score.^35^ This means years of education impact neuropsychological assessment results and, therefore, detection of cognitive decline. The results of other studies show that educational levels influence performance in the majority of analyzed tests regarding different cognitive domains^36^ and are also linked to functional disability and frailty.^37^ Some tests are not even designed to evaluate this population.^38^ The executive functioning performance also seems to be linked to educational level, as a community sample of Rio de Janeiro showed.^39^

One possible explanation is the influence of educational level in the formation of cognitive reserve. Cognitive reserve, formed by exposure to educational level and complex activities throughout life, would help resist neural damage.^40^ Because the educational level is usually higher in HIC, people would be more protected from cognitive decline than people of the same age in LMIC. In fact, findings show that successful aging predictors may be different between developing countries, such as Brazil, and HIC. In the former, socioeconomic status and social network structure may prevail over biological determinants,^41^ although a sixteen-year follow-up study showed that age and sex were the best predictors.^42^

Therefore, besides not being able to use the same methods in research and clinical settings as HIC, such as the use of biomarkers, LMIC older adults may also be exposed to unique adverse conditions during life, leading to different patterns in aging. An epidemiological study in São Paulo, Brazil and Buenos Aires, Argentina found higher rates of dementia in slums compared to developed countries.^7^ With all the difficulties presented in the screening for MCI, especially in LMIC, there is still one consensus: it is essential to diagnose pathological aging in its early stages when treatments work best.^43^ To do so, as seen, there is a need for screening tools in primary care that are fast and easy to administer and have high sensitivity and specificity for different backgrounds.^25^ It is still necessary to investigate the topic and search for ways to screen for MCI as a routine procedure during aging, especially in LMIC.

Ideally, this screening would be able to detect MCI in all its heterogeneity. While the cognitive criteria can be differently affected throughout all MCI types, the other MCI diagnosis criterion, functionality, might be more evenly affected. There is actually some controversy if ADL are intact or already impaired at the beginning of cognitive decline. A meta-analysis suggested the possibility of a continuum in the development of functional impairment, which would already begin to occur at MCI.^44^

But besides the discussion about whether there are already functional impairments in MCI, it is also important to address if individuals are aware of eventual impairments. If not, this would not be a complaint, so it could not be screened by self-report scales or be a variable for intervention planning. One example of functional deficit detection utility is an estimation that predicted a 10% reduction in life cost of individuals with dementia with an early intervention addressing functionality difficulties during one year.^5^ Assessing functionality when screening for MCI can thus be not only part of the diagnosis but also part of early intervention planning. Suppose these two variables can help to detect cognitive deficits at their early stages. In that case, their assessment will be a method that is fast, cheap, and amenable to a large-scale application - something even more critical in LMIC. After that, more complex and expensive examinations could be requested to differentiate between etiologies and indicate further treatments.

## ACTIVITIES OF DAILY LIVING

Besides having cognitive criteria, the original concept of MCI also required preserved functionality or ADL.^6^ ADL refer to tasks that are performed daily to maintain an independent life and a preserved functional capacity. It is usually divided into basic, instrumental, and advanced activities.^45^ ^,^ ^46^ Advanced ADL (aADL) are related to the most complex activities, such as maintaining hobbies or social life. Instrumental ADL (iADL) also have some complexity, for example, preparing meals and dealing with money, but refer to more day-to-day functional activities. Finally, basic ADL (bADL) have to do with self-care activities, such as bathing or eating. Impairment at any level can cause disability, and without the development of compensatory strategies to offset these difficulties, it can lead to dependence and decrease in the quality of life of people with dementia and their caregivers.^47^

At first, having deficits in ADL was the distinguishing criterion between MCI and dementia.^6^ But later evidence suggested that subtle changes already begin to occur in MCI, especially regarding complex activities.^44^ ^,^ ^45^ ^,^ ^48^ ^,^ ^49^ ^,^ ^50^ Another study^51^ proposed the inclusion of “preserved basic activities of daily living (ADL)/some minimal impairment in complex instrumental function” in the diagnostic process.

Since then, the literature questioning the presence or not of difficulties in everyday abilities in MCI groups has grown.^52^ ^,^ ^53^ ^,^ ^54^ Today, there are still no unified guidelines to diagnose MCI.^8^ However, it is known that there is a progressive loss of functional capacity in the course of dementia.^45^ ^,^ ^55^ ^,^ ^56^ But previous studies provide conflicting results about the extent to which functional capacity is affected at each moment of the condition.^54^ ^,^ ^57^ ^,^ ^58^ ^,^ ^59^ When cognitive decline reaches the threshold for the diagnosis of dementia, functionality is undoubtfully already compromised.^54^

## ACTIVITIES OF DAILY LIVING ASSESSMENT

There are many ways to measure functionality, and usually an instrument is focused on one level. Although scales are the most used tool, there are also performance-based tests and even in-home monitoring sensor technologies.^60^ Concerning scales, there are many options available to measure the different levels. Some examples widely used are the Lawton Instrumental Activities of Daily Living Scale developed by Lawton and Brody^61^ and the Pfeffer’s Functional Activities Questionnaire^62^ to measure iADL, the Katz Activities of Daily Living^63^ to measure bADL, and the Advanced Activities of Daily Living scale^64^ to measure aADL. Using report scales is the most convenient and practical way to measure ADL, and evidence shows that they are usually correlated with cognitive scores.^65^

But there are also downsides to this type of assessment. There is evidence indicating that informant- and self-report often differ substantially within dementia samples.^56^ ^,^ ^66^ ^,^ ^67^ Self-report also does not always correspond to objective measures of cognitive functioning,^68^ ^,^ ^69^ and there is considerable variability in the degree to which individuals with dementia and their caregivers differ regarding their report.^69^ ^,^ ^70^

One potential issue leading to the heterogeneity of results may be that individuals with dementia do not fully acknowledge the extent to which they have functional impairments. This lack of awareness about the diagnosis and its consequences, also termed anosognosia,^71^ is common in dementia.^72^ Although findings are mixed, it has been shown that people with MCI may also have limited awareness about their abilities.^73^ This could suggest that informant-based measures may be a better option when assessing functional abilities, but studies show that informants can underestimate abilities due to stress and caregiver burden.^74^

In this context, performance-based measures can provide more objective data than report scales.^57^ The assessment by performance-based measures requires various ADL activities to be performed in front of the examiner.^75^ However, direct measurements permit observation of only a small excerpt of real-world performance and are time-consuming.^76^ When compared to real-life monitoring, performance-based measures show different results.^60^ Other downsides that can influence individual performance are the novelty of the environment and even the evaluator’s presence.^60^

Examples of performance-based tools used to assess functional capacity in older adults are the UCSD Performance-based Skills Assessment (UPSA), whose results correlate with cognitive measures and evaluates five domains (comprehension and planning, finance, communication, transportation, and household chores),^77^ and the Revised Observed Test of Daily Living, which evaluates medication management, using the telephone and managing finances, but does not show a correlation with cognitive performance.^53^ All types of assessment have downsides and benefits, and the evaluator must be aware of them to choose the best option for a given objective and interpret the results correctly, whereas for diagnosis purposes or intervention planning.

## ACTIVITIES OF DAILY LIVING PERFORMANCE AWARENESS

One issue regarding measures that are not objective is that lack of awareness may produce an inaccurate result. Is it well known that individuals with dementia are usually not fully aware of their functional disabilities.^56^ Because of that, objective measures would be more recommended for this kind of patient. Nevertheless, ADL performance is assuredly compromised in dementia,^78^ so detecting functional impairment may be less of a challenge in this condition. When investigating MCI or another initial cognitive decline, it becomes more critical that the assessment is precise and detects subtle changes.^57^

Generally, studies investigating the topic use the comparison between self-report and informant-report, or self-report and performance-based measures, to determine if individuals with MCI are aware of their deficits.^56^ ^,^ ^65^ ^,^ ^74^ ^,^ ^79^ ^,^ ^80^ Both comparisons have advantages and disadvantages. While performance-based measures are more objective, they are not perfect when estimating real-life performance.^60^ On the other hand, comparisons to informant-report scales might be influenced by caregiver burden.^74^ Still, studies show its results are correlated with a patient’s cognitive function and are more accurate than self-reports.^65^ Findings are mixed and therefore need further investigation. This variation may be partially explained by some confounding variables, such as depression^80^ and cognitive level within MCI samples.^33^

## TYPES OF ERRORS IN ACTIVITIES OF DAILY LIVING PERFORMANCE

Some definitions can help deepen the topic. In general, evidence suggests that executive functioning is the best predictor of functional capacity.^45^ ^,^ ^58^ Still, there is also evidence that deterioration in the ability to perform everyday tasks could be related to a general cognitive impairment.^81^ To understand this difference, it is useful to differentiate between commission errors (performing a step incorrectly during a task) and omission errors (not performing an action at all). Evidence shows that only the latter error is related to a deficit in general cognitive resources.^81^ Besides general impairment, other studies found that omission errors also seem to be linked to memory impairment.^49^ ^,^ ^54^ Therefore, commission and omission errors would be different components of ADL impairment and could be uncorrelated since they depend on other cognitive domains.^81^

Regarding completing tasks, omission errors - not performing a step - might decrease the time spent on a task but might also prevent it from being completed correctly. Instead, commission errors can increase the time spent in an activity because a step is, for example, performed more than once or less properly because of executive function deficits.^81^ Therefore, the task can be completed, but in more time, because some types of errors generate longer responses, either because of a longer reaction time in the step of decision-making^82^ or because the individual needs more time to do the same task.^58^ If the time spent to complete an action is part of ADL performance decline, the awareness of this decline may also be related to cognitive performance. Looking at the literature concerning awareness of deficits, one study found that anosognosia is linked to memory impairment due to a lack of updating personal information.^83^ The results of another study showed that individuals with lower global cognitive performance overestimated their functional performance.^74^

Considering the latter cognitive correlates of unawareness and that only omission errors are related to global cognitive decline and memory impairment,^49^ ^,^ ^54^ ^,^ ^81^ only those would be linked to overestimating performance. Commission errors, linked to executive deficits, would still be perceived and reported by individuals during assessments because this type of cognitive impairment does not seem to be related to anosognosia.^80^ ^,^ ^84^

## STATE OF THE ART AND PROPOSAL

Diagnosis of MCI originally asked for: (1) cognitive complaint, (2) impaired cognition, and (3) preserved activities of daily living.^22^ As already discussed throughout this work, the last criterion has been questioned after evidence that there would already be some ADL impairment in people with MCI was brought to light. This concept might rely on different types of errors committed in the performance of ADL. There are commission errors (performing a step incorrectly - putting sugar twice in a recipe or baking it longer) that are related to executive functioning and hinder but do not prevent the execution of a task.^81^ And there are omission errors (not performing a step - not using sugar or baking at all), related to a deficit in general cognitive resources or memory and inability to complete the task.^49^ Also, studies investigating cognitive correlates of anosognosia or, even more specifically, ADL deficit awareness, show that the conditions are usually related to general cognitive decline^74^ or memory impairment.^83^

If omission errors are more related to general cognitive impairment or memory deficits,^49^ ^,^ ^54^ ^,^ ^81^ and if the same type of impairment is usually related to unawareness, it is therefore understandable that the error might not be perceived and, thus, reported. Alternatively, a commission error, where a step is performed with some difficulty and is linked to executive deficits rather than global deficits, might occur at the beginning of functional capacity decline, where global cognition is still preserved. These executive deficits do not seem to prevent perception by people involved.^80^ ^,^ ^84^

Further investigations are needed, but the evidence presented suggests that assessing functionality with attention to commission errors could be part of routine clinical screening tests for cognitive decline. It would be much more viable than recommended expensive alternatives that are not suitable for all health care systems.^12^ In other words, even if recent research has shown the possibility of using biomarkers and neuroimaging to detect neurodegenerative conditions in their prodromal or advanced stages,^85^ those options are not viable as a screening procedure, especially in LMIC. In the latter, neuropsychological assessment is a more accessible course of action,^12^ even in the initial stages.^31^ Activities of daily living scales are already used in clinical contexts, but a closer look at errors and their awareness could suggest the onset of a pathological process in the very beginning and be a useful approach to MCI screening.
